# Circumcision without Removing Any Part of Prepuce and under the Use of Cocaine

**Published:** 1891-09

**Authors:** L. G. Hardman

**Affiliations:** Harmony Grove, Ga.


### CIRCUMCISION WITHOUT REMOVING ANY PART OF PREPUCE AND UNDER THE USE OF COCAINE.

BY L. G. HARDMAN, M. D., HARMONY GROVE, GA.

I wish to call attention through your most valuable Journal to a most simple operation by the use of cocaine in circumcision. In the first place, it is an operation which should be done much more frequently than it is, and one reason why it is not is the objection to our anesthetics, such as chloroform or ether. Now, the operation which I prefer is the dorsal incision, which, when done, leaves the glands uncovered.

This is done with a groove director and scissors. The proove director is placed on the glands under the top of the prepuce, and pushed back to the corrona, and the incision is made then with the scissors in the groove director. You would think at first this would leave some corners on either side of the incision, but in fact it does not, but is relieved by the dorso-frunal contraction which, as it were, draws out the corners. Then the margin of the incision through the skin and mucous membrane turn toward each other, and they soon unite, even without section, but the section to bring the edges together will not be objectionable. Now, before you make your incision, inject a 40 per cent. solution of muriate of cocaine in the prepuce where you expect to make the incision, and the local anaesthetic effect of this agent will prevent any pain in the operation, and also the pain in the suturing the edges of the wound. This plan of operating makes the whole thing simple and not objectionable ; and so it can be done by any one without general anaesthesia. Therefore, it may be summed up as follows :

1st. Inject four per cent. solution of cocaine along the dorsum of the penis in the prepuce where you wish to make the incision.

2d. Introduce the groove director in the opening of the prepuce along the dorsum of the glands until you come back to the carroma glands. Then incise in groove director with scissors.

3d. Do not remove any part of the prepuce.

4th. If you use section, wait until the cocaine has bled out, as it were, from the prepuce before putting them in, and then you will not have any sloughing, otherwise you will save time.

This makes a very nice appearance when it is well.

